# 

**Published:** 1984-04

**Authors:** 


					Contents
Editorial
39.
Man and Medicine on Mendip
Norman Tricks
40.
Perianal abscess and its Recurrence
N. I. Ramus and J. Bradley
45.
Gilbert White: Medical and Social as-
pects of his letters
Philip Radford
46.
Surgeons and Surgery in Victorian
Yeovil
John R. Guy
48.
Prognostic Factors in Palindromic
Rheumatism
C. E. H. Grattan, T. D. Kennedy and
D. B. Yates
51
Clinical Diagnostic Accuracy in the
Management of Primary Stage 1 Cuta-
neous Malignant Melanoma in a Plastic
Surgery Unit
R. W. Griffiths, J. C. Briggs and
R. W. Hiles
55.
Obituary.
Dr. Colin Tribe
61.
From our Correspondents
64.
Surgical Club of South West England
67.
South West Orthopaedic Club
72.
continued overleaf
Book Review
74.
Correspondence
75.
75. Examination Results

				

